# Familial Mediterranean fever and COVID-19. An ancient disease in a pandemic of the new millennium: is it an epiphenomenon of infection?

**DOI:** 10.1093/rap/rkab097

**Published:** 2021-12-08

**Authors:** Timur S Sensoy, Richard Vollenberg, Jörn A Meier, Phil-Robin Tepasse

**Affiliations:** Department of Gastroenterology and Hepatology, University Hospital Munster, Munster, Germany

Key message

- Coronavirus disease 2019 and FMF could interact via pathological pathways; therefore, treatment should address both conditions.

Dear Editor, Coronavirus disease 2019 (COVID-19) is caused by severe acute respiratory syndrome coronavirus 2 (SARS-CoV-2). After transmission through inhaled droplets and aerosols, the virus enters the host cells by binding of its spike protein to the receptor for the angiotensin-converting enzyme 2 [1]. The host innate immune sensors recognize the pathogen-associated molecular patterns, and the first immune response occurs through the production of type 1 IFNs and the secretion of pro-inflammatory cytokines, including IL-6. Immune dysregulation could contribute to excessive pro-inflammatory signalling, including the induction of acute respiratory distress syndrome (ARDS), which has been associated with poor prognosis [2].

The autoinflammatory disease familial Mediterranean fever (FMF) is caused by mutations within the pyrin-encoding Mediterranean fever gene. The activation of the pyrin inflammasome leads to dysregulated pro-inflammatory cytokine production and inflammation [3]. It can be hypothesized that a hyper-responsive innate immune system might increase the risk of a severe course of COVID-19. Colchicine is the standard medication for the effective prophylactic treatment of inflammatory episodes and the prevention of reactive amyloidosis in FMF. Colchicine-resistant patients frequently receive anakinra or canakinumab [4]. IL-6 plays a pivotal role in FMF and is a promising new target [5]. Currently, studies are assessing the efficacy and safety of tocilizumab in FMF [6].

The German guidelines recommend tocilizumab, but not anakinra, in severe COVID-19 pneumonia, as of May 2021 [7]. However, there were no data available for immunosuppressed patients. Here, the first emergency administration of tocilizumab in an immunosuppressed patient with FMF and severe COVID-19 pneumonia with ARDS is described.

The patient was a 19-year-old female of Armenian origin, who was diagnosed with FMF and chronic kidney failure attributable to reflux nephropathy 16 years ago. The FMF attack frequency for this patient was zero to one episode per year under treatment. The patient received a kidney transplant 4 years ago and was immunosuppressed with prednisolone (5 mg/day), AZA (50 mg twice a day) and tacrolimus (2 mg twice a day). Proteinuria owing to chronic allograft nephropathy was present, although amyloidosis was ruled out in rectal biopsies that were carried out 15 and 7 years previously and in a renal allograft protocol biopsy that was carried out 1 year earlier. Colchicine treatment had occurred for 16 years and stopped 8 months before admission, owing to gastrointestinal side-effects. Anakinra had been used for 4 years because of colchicine-resistant FMF attacks.

Six days before admission (day 0), an episode of self-limiting abdominal pain without fever occurred. Owing to persisting dizziness and the onset of diarrhoea and nausea, a SARS-CoV-2 rapid antigen test was performed on day 3; the result was positive. She was not vaccinated and stopped anakinra owing to safety concerns. After developing a sore throat and dry cough, the patient was hospitalized on day 6. Upon examination, she was without fever (36.8°C) or dyspnoea (oxygen saturation of 98%). A chest X-ray revealed developing COVID-19 pneumonia. The initial investigations revealed a CRP of 13.8 mg/dl and IL-6 of 0.066 ng/ml. Procalcitonin was normal (0.13 ng/ml). The SARS-CoV-2 PCR test was positive for the B.1.1.7 variant.

On the morning of day 9, a second episode of abdominal pain and fever (38.6°C) occurred, followed by a decline in oxygen saturation to 83% some hours later. For adequate oxygenation, an oxygen mask with a flow of 5 l/min was initiated. A chest CT protocol showed that the patient had progressive COVID-19 pneumonia (Supplementary Fig. S1, available at *Rheumatology Advances in Practice* online). The laboratory results revealed a further elevation of CRP to 22.2 mg/dl and IL-6 to 1.016 ng/ml, while procalcitonin remained normal. Prednisolone was changed to dexamethasone, according to our standard operating procedures and guidelines [7]. After a further deterioration of respiratory function, 400 mg tocilizumab (body weight, 60 kg) was administered. The patient was positioned in an awake prone position, and high-flow oxygen was provided via a nasal cannula. The abdominal pain and fever resolved completely, and respiratory function remained stable after tocilizumab administration.

The investigations on day 11 revealed a decline in CRP to 13.7 mg/dl and an elevation of IL-6 to 13.835 ng/ml, which was most probably attributable to the saturation of the IL-6 receptors by tocilizumab; a consecutive decrease occurred in the following days [8]. The patient’s respiratory state eventually improved on day 14. The high-flow oxygen therapy ended on day 18, and the patient was discharged on day 23, after the normalization of respiratory function (Fig. 1 and Supplementary Table S1, available at *Rheumatology Advances in Practice* online).

**Fig. 1:**
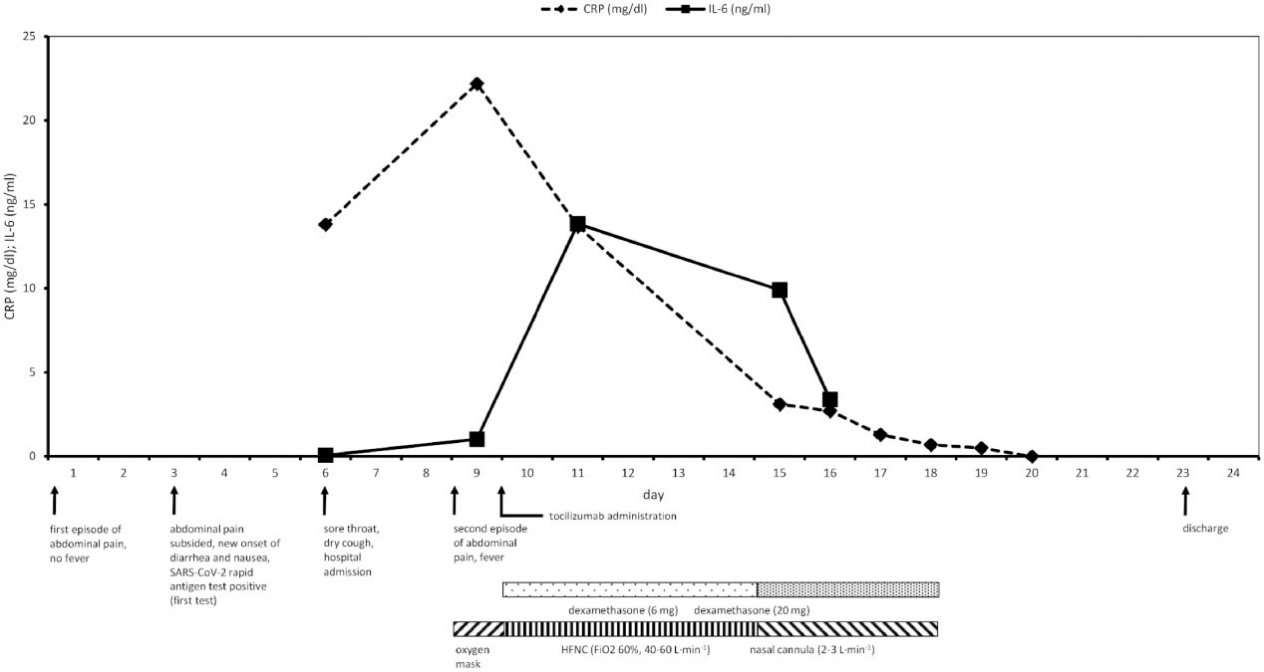
Pro-inflammatory markers, clinical features, medication and respiratory support HFNC: high-flow nasal cannula.

FMF and COVID-19 involve the activation of innate immune response mechanisms, and some of their clinical manifestations overlap, namely fever and abdominal pain. In contrast to COVID-19, FMF attacks are typically recurrent and are always self-limiting up to ∼72 h. In this case, the first episode of self-limiting abdominal pain (which was suggestive of FMF) occurred at the onset of COVID-19. A second episode of abdominal pain was followed by respiratory failure. This case is an addition to our understanding of how FMF and infectious diseases, such as COVID-19, interact if they occur in a patient simultaneously. It should be discussed whether the acquired infections are part of the pathogenesis of FMF. If so, therapy should also address the underlying causes. We used tocilizumab successfully to address both conditions.

*Funding:* No specific funding was received from any bodies in the public, commercial or not-for-profit sectors to carry out the work described in this manuscript.

*Disclosure statement:* The authors have declared no conflicts of interest.

*Consent:* Informed consent was provided for the publication of this manuscript.

## Data availability statement

The data that support the findings of this study are available on request from the corresponding author. The data are not publicly available due to privacy or ethical restrictions.

## Supplementary data

Supplementary data are available at *Rheumatology Advances in Practice* online.

## Supplementary Material

rkab097_Supplementary_Data
