# Supplementary material for: Familial Mediterranean fever and COVID-19. An ancient disease in a pandemic of the new millennium: is it an epiphenomenon of infection?
Source: Rheumatol Adv Pract. 2021 Dec 8;5(3):rkab097. doi: 10.1093/rap/rkab097 (PMC8717121; doi:10.1093/rap/rkab097)
Supplement: rkab097_Supplementary_Data [file rkab097_supplementary_data.docx]

**Supplementary Table S1:** **Proinflammatory markers.**

| Days since symptom onset | 6 | 9 | 11 | 15 | 16 | 17 | 18 | 19 | 20 |
| --- | --- | --- | --- | --- | --- | --- | --- | --- | --- |
| CRP (mg/dL) | 13.8 | 22.2 | 13.7 | 3.1 | 2.7 | 1.3 | 0.7 | 0.5 | < 0.5 |
| IL-6 (ng/mL) | 0.07 | 1.02 | 13.84 | 9.90 | 3.39 |  |  |  |  |
| Procalcitonin (ng/mL) | 0.13 | 0.16 | 0.21 | 0.22 | 0.14 |  |  |  |  |

CRP: C-reactive protein, IL-6: interleukin 6


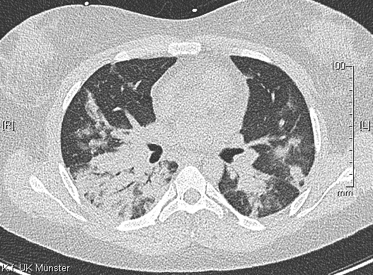
**Supplementary Figure S1:** **Low-dose chest computed tomography (day 9).**
